# Loss of significant association between high-sensitivity C-reactive protein (hs-CRP) and metabolic syndrome after adjustment for waist circumference found in 2022 Korea National Health and Nutrition Examination Survey data

**DOI:** 10.1186/s40101-025-00396-5

**Published:** 2025-06-05

**Authors:** Bo-Min Kim, So-Yeon Ryu, Mi-Ah Han, Seong-Woo Choi

**Affiliations:** 1 Department of Public Health, Graduate School of Health Science, Chosun University, Gwangju, Korea; 2 Department of Preventive Medicine, Chosun University Medical School, 20, Chosundae 4-gil, Dong-gu, Gwangju, 61452 Korea

**Keywords:** High-sensitivity C-reactive protein (hs-CRP), Components of metabolic syndrome, Adults in South Korea

## Abstract

**Background:**

Metabolic syndrome (MetS) is a cluster of metabolic abnormalities that increase the risk of cardiovascular disease and type 2 diabetes. High-sensitivity C-reactive protein (hs-CRP) is a biomarker of systemic inflammation, but its relationship with MetS and its components remains unclear. This study investigates the association between hs-CRP and MetS in a representative Korean population.

**Methods:**

Using data from the 2022 Korea National Health and Nutrition Examination Survey (KNHANES), we analyzed 4,823 adults. MetS was defined according to revised NCEP-ATP III criteria. Multivariate analyses were conducted, adjusting for confounders such as sex, age, income, education, smoking, alcohol consumption, physical activity, and waist circumference.

**Results:**

Among the study population, 1,784 participants (37.0%) were diagnosed with MetS. hs-CRP levels were significantly higher in individuals with MetS (1.06 mg/L vs. 0.79 mg/L, *p* < 0.001) and increased with the number of MetS components (p for trend < 0.001). Significant associations were observed between hs-CRP and all MetS components. However, after adjusting for waist circumference, these associations lost statistical significance.

**Conclusion:**

This study confirms a strong association between hs-CRP and MetS, primarily influenced by central obesity. The findings highlight abdominal obesity as a key contributor to systemic inflammation in MetS. Further longitudinal studies are needed to explore the causal relationship and underlying mechanisms.

## Introduction

Metabolic syndrome (MetS) is a cluster of metabolic abnormalities, including obesity, hypertension, hyperglycemia, and dyslipidemia, which significantly increase the risk of cardiovascular disease and type 2 diabetes. With the growing prevalence of sedentary lifestyles worldwide, the incidence of MetS is rising, making it a major contributor to increased mortality rates [1]. According to the Korean Society of Cardiometabolic Syndrome, the prevalence of MetS in Korea increased from 22.1% in 2007 to 24.9% in 2021 [2]. This trend in Korea parallels rapid modernization, urbanization, and changes in lifestyle patterns, including westernization of diets and decreased physical activity [3]. These sociocultural transitions may have contributed to the biological embedding of metabolic risk factors [4], such as abdominal obesity and systemic inflammation.

Inflammation initially serves as a defense mechanism against external stimuli. However, when chronic, it disrupts homeostasis and contributes to various diseases [5]. Chronic inflammation impairs metabolic function by increasing inflammatory cytokines, oxidative stress, and adipocyte-mediated inflammatory responses, which lead to insulin resistance and vascular damage, further exacerbating metabolic disorders [6].

Given the increasing global concern regarding MetS, organizations such as the World Health Organization (WHO) [7], the National Cholesterol Education Program Adult Treatment Panel III (NCEP ATP-III) [8, 9], and the International Diabetes Federation (IDF) [10] have established diagnostic criteria for MetS (Table 1). While these definitions commonly include cardiovascular risk factors, they differ in specific components and criteria.

**Table 1:** Diagnostic criteria for metabolic syndrome

Components	WHO (1999) [7]	NCEP-ATP III (2001) [8]	Revised NCEP-ATP III (2005) [9]	IDF (2005) [10]
Required criteria	Diabetes or impaired glucose tolerance or insulin resistance + at least two more of	Any three or more of	Any three or more of	Obesity + at least two more of
Obesity	BMI > 30 kg/m^2^ or WHR > 0.9 (M), > 0.85 (F)	WC ≥ 90 cm (M), ≥ 85 cm (F)^a^	WC ≥ 90 cm (M), ≥ 85 cm (F)^a^	WC ≥ 90 cm (M), ≥ 80 cm (F)^a^
TG	≥ 150 mg/dL	≥ 150 mg/dL	≥ 150 mg/dL or treatment	≥ 150 mg/dL or treatment
HDL-C	< 35 mg/dL (M), < 40 mg/dL (F)	< 40 mg/dL (M), < 50 mg/dL (F)	< 40 mg/dL (M), < 50 mg/dL (F) or treatment	< 40 mg/dL (M), < 50 mg/dL (F) or treatment
BP	≥ 140/90 mmHg or treatment	≥ 130/85 mmHg or treatment	≥ 130/85 mmHg or treatment	≥ 130/85 mmHg or treatment
FBG	Impaired glucose tolerance, IFG, or diabetes	≥ 100 mg/dL	≥ 100 mg/dL or treatment	≥ 100 mg/dL or diagnosed diabetes
Other	Microalbuminuria ≥ 20 μg/min	None	None	None

*WHO* World Health Organization, *NCEP-ATP III* National Cholesterol Education Program Adult Treatment Panel III, *IDF* International Diabetes Federation, *M* male, *F* female, *BMI* body mass index, *WHR* waist to hip ratio, *WC* waist circumference, *TG* triglycerides, *HDL-C* high-density lipoprotein cholesterol, *BP* blood pressure, *FPG* fasting plasma glucose

^a^WC standards were defined by the standards of the Korean Society for the Study of Obesity

High-sensitivity C-reactive protein (hs-CRP) is a liver-derived protein that serves as a biomarker for systemic inflammation [11, 12]. Recent studies have identified a strong association between elevated hs-CRP levels and MetS [13–16]. However, inconsistencies remain across studies due to differences in the definitions and assessment criteria for MetS. Specifically, the relationship between hs-CRP and individual MetS components varies across studies [15, 16].

Thus, this study aims to investigate the association between hs-CRP and MetS, as well as its individual components, in the general Korean population using data from the 2022 Korea National Health and Nutrition Examination Survey (KNHANES).

## Methods

### Study population

This study utilized raw data from the 2022 KNHANES, a nationwide cross-sectional survey conducted annually by the Korea Disease Control and Prevention Agency (KDCA) [17]. The KNHANES employs a complex, stratified, multistage probability-cluster sampling design to obtain a representative sample of non-institutionalized Korean citizens. It consists of three major components: a health interview, a health examination, and a nutrition survey. The KDCA Ethics Committee approved the study protocol (2018–01–03-4 C-A), and written informed consent was obtained from all participants.

A total of 6,265 individuals participated in the 2022 KNHANES, of whom 5,322 were adults aged 19 years or older. After excluding individuals with missing waist circumference or blood pressure (*n* = 269), missing hs-CRP data (*n* = 171), and suspected acute infections (hs-CRP ≥ 10 mg/L, *n* = 59), a final sample of 4,823 participants was analyzed.

### Measurements

#### Anthropometric characteristics

Anthropometric variables included sex, age, income, education, marital status, smoking, alcohol consumption, and physical activity. Age was categorized into three groups: 19–39 years, 40–64 years, and ≥ 65 years. Income levels were classified into quartiles based on monthly household income. Education level was divided into three categories: middle school or lower, high school graduate, and college graduate or higher. Marital status was categorized as married or unmarried. Smoking status was classified as smoker or non-smoker, and alcohol consumption was classified as drinker or non-drinker. Physical activity was categorized as active if participants engaged in at least 150 min of moderate-intensity activity or 75 min of vigorous-intensity activity per week; otherwise, they were classified as inactive.

#### Metabolic syndrome definition

MetS was defined according to the revised NCEP-ATP III criteria [9], incorporating central obesity cut-offs from the Korean Society for the Study of Obesity [18]. A diagnosis of MetS required the presence of three or more of the following components: (1) Waist circumference ≥ 90 cm for men and ≥ 85 cm for women; (2) Triglycerides ≥ 150 mg/dL or treatment for hypertriglyceridemia.; (3) HDL-C < 40 mg/dL in men or < 50 mg/mL in women or treatment for low HDL-C; (4) Blood pressure ≥ 130/85 mmHg or treatment for hypertension; and (5) Fasting glucose ≥ 100 mg/dL or treatment for hyperglycemia..

#### hs-CRP and laboratory analysis

Blood samples were collected after an overnight fast (≥ 8 h) and stored in EDTA-coated tubes. Serum samples were refrigerated at 2–8 °C, and all laboratory tests were conducted within 24 h of collection. hs-CRP levels were measured using an immunoturbidimetric assay (Cobas, Roche, Germany), with daily calibration against reference standards. Other biochemical markers, including fasting blood glucose, total cholesterol, triglycerides, and HDL cholesterol, were measured using an automated biochemical analyzer (TOSHIBA Accute 80 FR, Japan). Systolic and diastolic blood pressure measurements were taken three times, and the final values were derived from the average of the second and third readings.

#### Data analysis

All values are given as a number (percentage) or mean ± standard deviation. The chi-square test (χ^2^-test) was used for categorical variables, while independent t-tests were used for continuous variables to compare based on MetS status. Analysis of covariance (ANCOVA) was conducted to compare mean hs-CRP values across MetS status and components. The Model I was adjusted for sex and age, and Model II was further adjusted for income, education, martital status, current smoking, drinking, physical activity. Model III was additionally adjusted for waist circumference. Statistical analyses were conducted using SPSS (ver. 29.0; IBM, Armonk, NY, USA), with statistical significance set at *P* < 0.05.

## Results

### Prevalence of MetS and its components

The prevalence of MetS and its components is presented in Table 2. Among the 4,823 participants, 1,784 (37.0%) were diagnosed with MetS. As shown in Table 2, when classified by the number of MetS components, 1,327 (27.5%) had none, 899 (18.6%) had one, 813 (16.9%) had two, 819 (17.0%) had three, 604 (12.5%) had four, and 361 (7.5%) had all five components. Regarding individual MetS components, the most prevalent was hypertension (41.8%), followed by hypertriglyceridemia (40.6%), high blood glucose (37.6%), low HDL cholesterol (35.9%), and central obesity (34.8%).

**Table 2:** Prevalence of metabolic syndrome and its components (*N* = 4823)

Category	N (%)
Metabolic syndrome	1,784 (37.0)
Number of metabolic syndrome components	
0	1,327 (27.5)
1	899 (18.6)
2	813 (16.9)
3	819 (17.0)
4	604 (12.5)
5	361 (7.5)
Prevalence of each component	
Central obesity	1,680 (34.8)
Hypertension	2,017 (41.8)
High blood glucose	1,814 (37.6)
Hypertriglyceridemia	1,960 (40.6)
Low HDL cholesterol	1,732 (35.9)

### Characteristics between MetS and non-MetS

The baseline characteristics of participants stratified by MetS status are detailed in Table 3. Among all 4,823 participants, 2,098 (43.5%) were men and 2,725 (56.5%) were women. The age distribution was as follows: 1,166 participants (24.2%) were aged 19–39 years, 2,148 (44.5%) were aged 40–65 years, and 1,509 (31.3%) were aged 65 years or older. As presented in Table 3, the MetS group had significantly higher proportions of older age, male sex, lower education, and lower income (p < 0.001). Additionally, mean waist circumference, systolic/diastolic blood pressure, fasting glucose, triglycerides, and hs-CRP levels were significantly higher in the MetS group. Conversely, HDL cholesterol, physical activity levels, and alcohol consumption were significantly lower (all p < 0.001).

**Table 3:** Characteristics according to metabolic syndrome

Variable	Total	Metabolic syndrome	Non-metabolic syndrome	*P*-value
Sex				< 0.001
Men	2,098 (43.5)	878 (49.2)	1220 (40.1)	
Women	2,725 (56.5)	906 (50.8)	1819 (59.9)	
Age (years)				< 0.001
< 40	1,166 (24.2)	137 (7.7)	1029 (33.9)	
40–64	2,148 (44.5)	754 (42.3)	1394 (45.9)	
≥ 65	1,509 (31.3)	893 (50.1)	616 (20.3)	
Income				< 0.001
Lowest	999 (20.7)	523 (29.3)	476 (15.7)	
Mid-low	1,163 (24.2)	482 (27.0)	681 (22.5)	
Mid-high	1,299 (27.0)	413 (23.2)	886 (29.2)	
Highest	1,354 (28.1)	364 (20.4)	990 (32.6)	
Education				< 0.001
Middle school graduate or lower	1,214 (26.0)	716 (42.4)	498 (16.8)	
High school graduate	1,550 (33.3)	504 (29.9)	1046 (35.2)	
College graduate or higher	1,895 (40.7)	468 (27.7)	1427 (48.0)	
Marital status				< 0.001
Yes	3,193 (66.2)	1254 (70.3)	1939 (63.8)	
No	1,630 (33.8)	530 (29.7)	1100 (36.2)	
Current smoking	712 (14.9)	297 (16.9)	415 (13.7)	0.003
Drinking	4,287 (89.5)	1491 (84.6)	2796 (92.3)	< 0.001
Physical activity	2,082 (46.4)	619 (37.8)	1463 (51.3)	< 0.001
Waist circumference (cm)	84.1 ± 10.8	90.9 ± 9.3	80.0 ± 9.4	< 0.001
Systolic blood pressure (mmHg)	119.9 ± 16.3	127.9 ± 16.5	115.3 ± 14.3	< 0.001
Diastolic blood pressure (mmHg)	74.2 ± 9.8	77.0 ± 10.2	72.5 ± 9.2	< 0.001
Blood glucose (mg/dL)	101.3 ± 21.3	112.3 ± 27.1	94.8 ± 13.1	< 0.001
Total cholesterol (mg/dL)	188.7 ± 40.6	178.2 ± 43.8	194.8 ± 37.3	< 0.001
Triglyceride (mg/dL)	127.8 ± 92.7	169.6 ± 122.8	103.2 ± 56.1	< 0.001
LDL cholesterol (mg/dL)	114.7 ± 37.1	104.2 ± 39.4	120.8 ± 34.2	< 0.001
HDL cholesterol (mg/dL)	57.4 ± 15.3	50.5 ± 13.6	61.4 ± 14.8	< 0.001
Hs-CRP (mg/L)	0.88 ± 1.23	1.20 ± 1.38	0.75 ± 1.14	< 0.001

### Association of hs-CRP with MetS and its components

The association between hs-CRP levels and MetS is summarized in Table 4. After adjusting for sex, age, and other covariates (Model II), mean hs-CRP levels significantly increased with the number of MetS components (p for trend < 0.001, see Table 4). Participants with MetS had higher mean hs-CRP levels (1.06 [1.00–1.13] mg/L) than those without MetS (0.79 [0.74–0.83] mg/L, p < 0.001). As shown in Table 4, hs-CRP levels were also significantly higher among individuals with each component of MetS, including central obesity (1.18 vs. 0.73), hypertension (1.01 vs. 0.80), high blood glucose (1.01 vs. 0.82), hypertriglyceridemia (0.96 vs. 0.84), and low HDL cholesterol (1.01 vs. 0.82) (all p < 0.001). However, these associations lost statistical significance after additional adjustment for waist circumference (Model III).

**Table 4:** The asssociation of hs-CRP with metabolic syndrome and its components

Variable	Model I	Model II	Model III
Number of metabolic syndrome component			
0	0.53 (0.46–0.59)	0.52 (0.45–0.59)	0.75 (0.67–0.83)
1	0.79 (0.72–0.86)	0.80 (0.73–0.87)	0.87 (0.80–0.94)
2	0.98 (0.91–1.06)	0.99 (0.91–1.07)	0.92 (0.84–1.00)
3	1.13 (1.05–1.21)	1.12 (1.04–1.21)	0.92 (0.83–1.01)
4	1.37 (1.25–1.49)	1.34 (1.22–1.47)	1.06 (0.92–1.19)
5	1.45 (1.23–1.67)	1.44 (1.21–1.66)	1.09 (0.86–1.31)
P for trend	< 0.001	< 0.001	0.009
Metabolic syndrome			
No	0.72 (0.67–0.76)	0.76 (0.72–0.80)	0.85 (0.81–0.89)
Yes	1.18 (1.12–1.24)	1.19 (1.12–1.26)	0.94 (0.86–1.01)
*P*-value	< 0.001	< 0.001	0.067
Central obesity			
No	0.72 (0.67–0.76)	0.71 (0.67–0.76)	
Yes	1.18 (1.12–1.24)	1.18 (1.11–1.24)	
*P*-value	< 0.001	< 0.001	
Hypertension			
No	0.78 (0.73–0.83)	0.78 (0.74–0.83)	0.86 (0.81–0.91)
Yes	1.01 (0.95–1.07)	1.00 (0.94–1.06)	0.90 (0.84–0.96)
*P*-value	< 0.001	< 0.001	0.359
High blood glucose			
No	0.79 (0.75–0.84)	0.80 (0.75–0.84)	0.86 (0.81–0.90)
Yes	1.01 (0.95–1.07)	1.00 (0.94–1.06)	0.90 (0.84–0.96)
*P*-value	< 0.001	< 0.001	0.279
Hypertriglyceridemia			
No	0.80 (0.76–0.84)	0.80 (0.75–0.84)	0.84 (0.80–0.88)
Yes	1.11 (1.04–1.18)	1.11 (1.03–1.18)	0.98 (0.90,1.05)
*P*-value	< 0.001	< 0.001	0.001
Low HDL cholesterol			
No	0.79 (0.75–0.83)	0.79 (0.75–0.83)	0.82 (0.78–0.86)
Yes	1.22 (1.15–1.30)	1.21 (1.13–1.29)	1.08 (1.00,1.16)
*P*-value	< 0.001	< 0.001	< 0.001

Values are presented as mean (95% CI)

Model I is adjusted by sex and age

Model II is adjusted by Model I plus income, education, marital status, current smoking, driking, and physical activity

Model III is adjusted by Model II plus waist circumference

## Discussion

This study investigated the relationship between hs-CRP and MetS using data from the 2022 KNHANES. The results demonstrated that after adjusting for sex, age, and other covariates, hs-CRP levels were significantly higher in participants with MetS and in all individual components of MetS. However, after further adjustment for waist circumference, the significant association between hs-CRP and MetS disappeared. These findings should be interpreted within the broader biocultural context of Korea's rapid modernization, urbanization, and lifestyle transitions [3]. In particular, changes such as dietary westernization and reduced physical activity have likely contributed to systemic inflammation and metabolic dysregulation [19]. Thus, the biological patterns observed in this study may reflect the embodiment of sociocultural and environmental transformations [20], highlighting how modern societal changes influence health outcomes [21].

The mean hs-CRP level in this study was significantly higher in individuals with MetS, which aligns with findings from previous studies [22–25]. For instance, a study conducted in Iran on a general population of 7,284 individuals reported significantly higher hs-CRP levels in those with MetS [23]. Similarly, a study in Portugal involving 1,022 participants found the mean hs-CRP level (95% CI) was 2.34 (1.97–2.78) mg/L in individuals with MetS, compared to 1.36 (1.26–1.47) mg/L in those without MetS, demonstrating a significant difference (*p* < 0.001) [24]. Furthermore, our study found that hs-CRP levels increased with the number of MetS components, a trend also observed in a study using data from the 2009–2010 NHANES, which reported that the odds ratio for high-risk hs-CRP (> 3 mg/dL) increased as the number of MetS components grew [25].

Since the concept of Syndrome X evolved into MetS, ongoing efforts have focused on defining its related components for clinical practice. Recently, central obesity has been highlighted as a key factor influencing other MetS components, such as blood pressure, blood glucose, and dyslipidemia. Central obesity, measured by waist circumference, has been identified as a critical factor due to its strong association with cardiovascular disease and other MetS components. It is considered an early step in the pathogenesis of MetS [26]. The International Diabetes Federation (IDF) even designates abdominal obesity as an essential criterion for diagnosing MetS, requiring the presence of abdominal obesity along with at least two additional components [10]. Previous studies on the relationship between hs-CRP and MetS have similarly identified obesity as a major determinant of elevated CRP levels [14]. Our findings support this, as the additional adjustment for waist circumference was conducted as an exploratory attempt to assess the mediating role of central obesity in the association between hs-CRP and MetS, underscoring abdominal obesity as the primary determinant of this relationship.

The underlying mechanism involves adipose tissue, which plays a significant role in releasing cytokines such as interleukin-6 (IL-6) and tumor necrosis factor-α (TNF-α) [27]. Furthermore, macrophage infiltration of adipose tissue in obese individuals contributes to systemic inflammation, particularly in cases of abdominal obesity [28]. The liver, the primary source of hs-CRP, synthesizes hs-CRP mainly in response to IL-6 and other cytokines, with about 30% of circulating IL-6 originating from adipose tissue in healthy individuals [29].

Our study also found significant associations between hs-CRP and all individual components of MetS. A study in Israel with 1,929 participants reported significant associations between CRP and all MetS components [14], which is consistent with our results. However, some previous studies have reported inconsistent findings. For example, a study in Portugal [24] found no association between hs-CRP and HDL cholesterol, while a study in Italy [16] reported no significant association with triglycerides or blood pressure. Similarly, a study in Germany [30] found no significant association between hs-CRP and triglycerides, blood pressure, or HDL cholesterol. These discrepancies may stem from differences in the definitions of MetS across studies. Various criteria, including WHO, ATP III (2001), revised ATP III (2005), and IDF, have established different definitions for MetS (Table 1), leading to variations in prevalence and associations with other variables, even within the same study population. In our study, the prevalence of MetS was 27.8% using the ATP III (2001) criteria, but increased to 37.0% when applying the revised ATP III (2005) criteria, showing a nearly 10% difference based on the definition used.

### Strength and limitations

This study has several limitations. First, due to its cross-sectional design, we can only confirm an association between hs-CRP and MetS but cannot establish causality. Longitudinal studies are needed to further elucidate temporal and causal relationships. Second, the hs-CRP measurements exhibited a large standard deviation relative to the mean, and the number of significant figures was limited (only one to two digits). This high variability limits the clinical applicability of hs-CRP as a diagnostic biomarker for MetS at the individual level. While hs-CRP may serve as a useful population-level marker of systemic inflammation, it should not be used alone to classify individuals into MetS risk categories. Third, potential residual confounding may exist due to unmeasured factors such as medication use for hypertension, diabetes, or dyslipidemia. Despite these limitations, this study contributes valuable insights into the association between systemic inflammation and metabolic dysregulation in a rapidly modernizing society.

## Conclusion

This study demonstrates a strong association between hs-CRP levels and MetS, with central obesity appearing to play a major mediating role in this relationship. The findings suggest that abdominal obesity is an important factor associated with systemic inflammation in individuals with MetS. Further longitudinal studies are needed to elucidate the temporal sequence and causality underlying these associations.

## Data Availability

Data are available from the Korea National Health and Nutrition Examination Survey database (https://knhanes.kdca.go.kr/knhanes/rawDataDwnld/rawDataDwnld.do).
